# Occurrence Regionalization of Kiwifruit Brown Spot in Sichuan

**DOI:** 10.3390/jof9090899

**Published:** 2023-08-31

**Authors:** Yuhang Zhu, Kaikai Yao, Miaomiao Ma, Yongliang Cui, Jing Xu, Wen Chen, Rui Yang, Cuiping Wu, Guoshu Gong

**Affiliations:** 1 Plant Protection Department, College of Agronomy, Sichuan Agricultural University, Chengdu 611130, China; s20163305@stu.sicau.edu.cn (Y.Z.); yaokaikai@stu.sicau.edu.cn (K.Y.); 14915@sicau.edu.cn (M.M.); b20171803@stu.sicau.edu.cn (J.X.); 2020301154@stu.sicau.edu.cn (W.C.); 2020201073@stu.sicau.edu.cn (R.Y.); 71262@sicau.edu.cn (C.W.); 2 Sichuan Provincial Academy of Natural Resource Sciences, Chengdu 610041, China; yongliangcui@163.com

**Keywords:** kiwifruit brown spot, *Corynespora cassiicola*, MaxEnt, GIS, environmental variables, epidemiology

## Abstract

Kiwifruit brown spot caused by *Corynespora cassiicola* is the most significant fungal disease in Sichuan, resulting in premature defoliation, which had a significant impact on yield and fruit quality. The objective of the study was to determine the occurrence regularity and suitability of kiwifruit brown spot in Sichuan. The occurrence of the disease in the main producing region was continuously monitored, the maximum entropy (MaxEnt) model was used to predict its potential distribution, and the key environmental variables were identified using the jackknife method. The results indicated that kiwifruit brown spot was widely distributed across the entire producing region in Sichuan, predominantly affecting the variety “Hongyang”. The incidence (*p* < 0.01) and disease index (*p* < 0.05) showed a significant positive correlation with the cultivar, and decreased with the altitude increasing. The average area under the ROC curve (AUC) of 10 replicates was 0.933 ± 0.012, with an accuracy of 84.44% in a field test, confirming the reliability of the predicted results. The highly suitable distribution areas of kiwifruit brown spot were mainly located in the Chengdu and Ya’an regions. The entire Panzhihua region was an unsuitable distribution area, and the entire Pujiang County and Mingshan District were highly suitable distribution areas. The key environmental variables affecting the potential distribution of kiwifruit brown spot included isothermality (24.3–33.7%), minimum temperature in August (16.3–23.6 °C), maximum temperature in July (25.5–31.2 °C), minimum temperature in June (15.6–20.9 °C), precipitation in August (158–430 mm), and average temperature in October (15.6–18.8 °C). This study provides a theoretical basis for the reasonable layout of the cultivar and the precise prevention and control of the disease.

## 1. Introduction

Kiwifruit is one of the most popular fruits worldwide due to its special flavor and abundant nutrient contents, especially vitamin C, minerals, and dietary fiber [1,2]. Kiwifruit is a perennial cash crop, which can provide continuous considerable economic benefit to growers [3]. At present, kiwifruit occupies an important position in the international fruit business. In leading producing countries, such as China, New Zealand, and Italy, kiwifruit is the main source of income for local families, making it a vital parameter in their economy [4]. The kiwifruit industry in Sichuan has experienced unprecedented development since 2008, with its current output ranking second in China [5]. Among the cultivated varieties, red-fleshed kiwifruit, especially “Hongyang”, has been widely cultivated, with a planting area reaching 50,000 hectares until 2018. Kiwifruit brown spot caused by *Corynespora cassiicola* is a fungal airborne disease [6,7], which is the second most significant disease, followed by kiwifruit bacterial canker disease, in Sichuan [8]. The disease spreads rapidly, and the incidence can reach 90% to 100% during the harvest stage. In some orchards, functional leaves are almost entirely lost, leading to the premature sprouting of autumn shoots, and the fruits lose water severely, shrivel, and soften, losing commodity value and causing huge yield loss [8]. With the continuous cultivation of highly susceptible varieties, kiwifruit brown spot has become an escalating concern and a significant limiting factor for industry development. Therefore, understanding the occurrence regularity and distribution regionalization of the disease is of utmost importance.

Previous studies on the epidemic of plant diseases and pests have been limited by technical barriers and other factors, restricting their spatial development to relatively small scales, typically within a confined infected field. However, recent advancements in geographic information system (GIS) technology have enabled the analysis of large-scale spatial data, which makes it possible to regionalize the occurrence and development regularity of diseases and pests in large-scale regions, even on a global scale. Zou et al. [9] regionalized the oversummer range of wheat powdery mildew using the GIS ordinary kriging method combined with the digital elevation model (DEM) of China. Kistner-Thomas et al. [10] assessed the relationship between grasshopper density survey data and 72 biologically relevant GIS-based environmental variables and developed a regression model to predict the mean density of an adult grasshopper from 2012 to 2016. At present, in the research on the suitable regionalization of diseases and pests, correlation analysis, weight coefficient distribution, and assignment of each impact factor are first carried out with mathematical methods; then the comprehensive factors are graded by the index classification method, the data are rasterized by GIS, the value of the vacant area is inserted, and the regionalization results are finally determined [11]. The accuracy of the model primarily relies on the key factors. Accurately understanding occurrence regularity could lay a good foundation for the subsequent distribution regionalization. Peng et al. [12] successfully identified the occurrence and epidemic regularity of wheat stripe rust in Nanchong, achieving 100%, 98%, and 95% accuracy rates for short-term, medium-term, and long-term predictions, respectively. Chen et al. [13] used trajectory analysis and an effective accumulated temperature model to simulate the migration process and development progress of *Spodoptera frugiperda*, clarifying its migration path and occurrence regionalization in China.

The maximum entropy theory was the most objective criterion for selecting the statistical characteristics of random variables. Based on this theory, the maximum entropy (MaxEnt) model is a quantitative analysis tool, which has been widely applied in plant protection due to its stable operation, simplicity, rapid calculation, and high accuracy [14,15,16]. Zhang et al. [17] collected geographic location information on *Prunus salicina* and one of the brown rot pathogenic species (*Monilinia fructicola*), applying the MaxEnt model to simulate their potential suitable distribution in China. Wang et al. [18,19] utilized the MaxEnt model based on distribution information and environmental variables to investigate the suitability of kiwifruit bacterial canker disease in Sichuan and predict its potential distribution under climate change. Evaluating the accuracy of the model is an essential step, accomplished by employing different evaluation methods and standards with effective evaluation indexes. Among these, the area under the receiver operating characteristic (ROC) curve (AUC) has been widely used. Wei et al. [20] used the AUC to evaluate the accuracy of the MaxEnt model in predicting the potential distribution of maize chlorotic mottle virus (MCMV) under historical and future climatic conditions. Cho et al. [21] evaluated the accuracy of spatial (regression kriging) and nonspatial (MaxEnt) models to simulate the distribution of two invasive plant species (*Ambrosia artemisiifolia* and *Ambrosia trifida*) by AUC.

Kiwifruit brown spot primarily occurs during high temperature and high humidity seasons, and initially appears in late June and lasts until October. On the other hand, kiwifruit bacterial canker disease is a low-temperature disease, occurring from November to May the following year. Both diseases significantly threaten the healthy development of the kiwifruit industry and substantially impact the variety layout. Meanwhile, their phenological periods and the physiological and biochemical characteristics are completely different. Therefore, the development of this study can provide more scientific and rational guidance for the variety layout of kiwifruit in Sichuan. At present, the occurrence regularity of kiwifruit brown spot in Sichuan has not been clearly elucidated, resulting in blind prevention and control and serious pesticide abuse in production, which is not conducive to the healthy development of the industry. The international scientific community has dedicated many efforts to enhancing resilience and sustainability in agriculture, with a particular emphasis on reducing pesticides [22], including in kiwifruit cultivation [23,24]. Disease prediction is a prerequisite for disease management and plays an important role in integrated pest management [25]. The database established based on long-term monitoring is the foundation for an accurate disease prediction [26]. Early preparation for prevention can not only improve the effectiveness and benefits, but also reduce unnecessary costs and environmental pollution caused by pesticide abuse [27]. Moreover, studies on kiwifruit brown spot are still in the initial stage and mainly focus on pathogen identification, population diversity, resistance of cultivars, and disease control method [3,6,7,28]. However, a suitable regionalization based on climate conditions remains unknown, warranting further study.

In this study, the epidemic of kiwifruit brown spot in the main producing region was monitored over the long term, and the potential distribution of this disease in Sichuan was employed to predict it using the MaxEnt model. The objectives of this study were to (1) clarify the disease occurrence regularity to provide rational guidance for the variety layout; (2) to evaluate the key environmental variables affecting the distribution of this disease; (3) to determine the suitability of this disease in Sichuan, aiming to provide a theoretical basis for the prediction, precise prevention, and control of the disease.

## 2. Materials and Methods

### 2.1. Sources of Software and Map

MaxEnt software (version 3.4.1) was downloaded from the Museum of Natural History (https://biodiversityinformatics.amnh.org/open_source/maxent/, accessed on 29 November 2022), Java software was downloaded from its official website (https://www.oracle.com/java/, accessed on 31 July 2022), ArcGIS software (version 10.8.1) was downloaded from the ESRI website (https://support.esri.com/en/Products/Desktop/arcgisdesktop/arcmap, accessed on 1 December 2022), and the base map was provided by the National Meteorological Information Centre of China.

### 2.2. Determination of the Occurrence of Kiwifruit Brown Spot

During 2012–2022, the occurrence and distribution of kiwifruit brown spot in the main production regions in Sichuan were continuously assessed. Detailed geographical information (longitude, latitude, and altitude), cultivar, and disease occurrence were recorded. The assessment involved six prefecture-level administrative regions, including Chengdu, Ya’an, Guangyuan, Deyang, Mianyang, and Meishan, and was conducted at the end of the disease logistic phase, namely, mid- to late September.

### 2.3. Measurement of Disease Severity

Each experimental site consisted of five plots, with three trees per plot, totaling fifteen trees. Each tree was examined from five directions (east, west, south, north, and middle), with two branches per direction. From each branch, five leaves were selected, starting from the base upwards, resulting in a total of fifty leaves per tree. The standard of classification for disease severity is presented in Table 1. The disease index (DI) was calculated according to the following formula [3]. The disease level was divided into three categories [8]: high (DI ≥ 66.67), moderate (33.33 ≤ DI < 66.67), and low (0 < DI < 33.33).

$$DI=\frac{\sum \left(the\;{leaves}^{\prime }number\;of\;each\;severity\times severity\right)}{the\;number\;of\;leaves\times the\;highest\;severity}\times 100$$

### 2.4. Correlation Analysis

The Pearson correlation between variety and disease index was determined by SPSS 21.0. The altitude and disease index of the “Hongyang” variety were extracted for correlation analysis.

### 2.5. Acquisition and Processing of Distribution Information

Kiwifruit brown spot disease primarily occurred in red-fleshed kiwifruit, widely cultivated in Sichuan. It has also been reported in Guangxi, Hubei, and Chongqing, exhibiting strong regional characteristics. Therefore, the other distribution information was mainly obtained by searching published papers [3,7,28], inquiring the kiwifruit disease and pest prediction and forecast reports issued by local plant protection stations and agricultural technology and meteorological cooperative service reports, and consulting local plant protection staff. Through the above procedure, 108 occurrence records were evaluated. By incorporating the 122 available experimental sites collected during the initial stages, a total of 230 distribution points were obtained.

**Figure 1:**
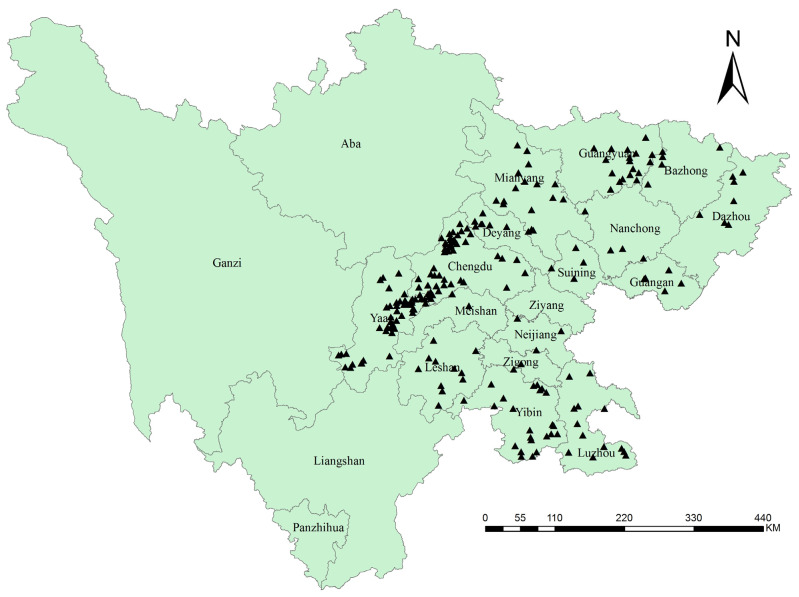
Sample distribution points of kiwifruit brown spot.

The distribution points with specific information were directly applied, while those lacking latitude and longitude were inquired in the global geographic information integrated database GeoName and the Baidu coordinate picking system to extract the coordinate information. The buffering area analysis function of ArcGIS was used to calculate the distance between the grid center and distribution points. Only the closest record to the center was retained within the same grid. The above distribution records in the order of species, longitude, and latitude were imported into Excel, with a positive north latitude and east longitude and a negative south latitude and west longitude. After the screening process, 225 valid points were retained for constructing the models (Figure 1), and the species distribution information was transferred to the CSV file required by MaxEnt.

### 2.6. Acquisition and Processing of Environmental Variables

All environmental data were obtained for free from the WorldClim database (http://www.worldclim.org/, accessed on 28 August 2023), which provides interpolated raster data based on global meteorological record information. The above data were in TIFF format with a spatial resolution of 2.5 arc-minutes, including 67 environmental variables, among which were 19 bioclimatic factors, monthly mean precipitation, monthly mean temperature, maximum and minimum temperature, and other bioclimatic indices (Table 2). The environmental variables were extracted from the administrative zoning map of Sichuan as the base map. The TIFF format was transferred to the ASCII format required by MaxEnt using the format conversion function of ArcGIS. Initially, 67 environmental variables were extracted to build an initial model. The contribution rates and importance of environmental variables were determined by the jackknife method, and those rates of less than 1% were eliminated. The attribute values of environmental variables at 225 distribution points were extracted using the extraction analysis tool of ArcGIS. Pearson correlations between environmental variables were calculated using SPSS 21.0. Variables with a strong correlation were removed, and the relationship between kiwifruit brown spot and meteorological factors was considered to finally screen out variables.

### 2.7. Construction and Evaluation of MaxEnt Model

The distribution information of kiwifruit brown spot and environmental variables were imported into MaxEnt, and the climate response curve was created to analyze the relationship between each environmental variable and the distribution probability. The prediction map was drawn, and the importance of environmental variables was measured using the jackknife method. The random test percentage was set to 25% and repeated 10 times. The default values of the model were selected for other parameters, and the output path was set for modeling. In this study, AUC was used to evaluate the accuracy of the model simulation. The AUC value ranges from 0.5 to 1, with values closer to 1 indicating a stronger correlation between environmental variables and species distribution and a higher model accuracy. The evaluation criterion is 0.5 ≤ AUC < 0.6, fail; 0.6 ≤ AUC < 0.7, poor; 0.7 ≤ AUC < 0.8, general; 0.8 ≤ AUC < 0.9, good; AUC ≥ 0.9, excellent.

### 2.8. Geographic Division of Suitability

The ASCII format files output by MaxEnt were transferred to raster format files using the format conversion function of ArcGIS. The potential distribution map of kiwifruit brown spot was extracted from the administrative zone map of Sichuan as the base map, and then the spatial analysis tool was used for reclassification. According to previous studies [29,30], the suitable area was divided into four categories, displaying them in different colors: highly suitable area (*p* > 0.66, red), moderately suitable area (0.33 < *p* ≤ 0.66, orange), lowly suitable area (0.05 < *p* ≤ 0.33, yellow), and unsuitable area (*p* ≤ 0.05, white). The distribution area of each region and district (county) was calculated using the statistical analysis function of ArcGIS.

### 2.9. Field Evaluation of the Model

A field test of species distribution was conducted as the most direct and reliable method for model validation. In order to further verify the accuracy of the simulation results, additional 45 actual occurrence records from supplementary determination were introduced for the field test. The actual distribution points were mapped onto the reclassified map, and the corresponding suitable levels were extracted by ArcGIS, and then compared with the actual level of disease occurrence. Both are equally regarded as accurate.

## 3. Results

### 3.1. Occurrence of Kiwifruit Brown Spot

A long-term monitoring of the epidemic of kiwifruit brown spot in the main production region revealed that the disease was widely distributed and seriously occurred in all regions (Table 3). Out of 122 investigation sites in the province, 83 sites (68.03%) exhibited a high occurrence level. The disease occurrence was particularly severe in the main cultivated variety, ‘Hongyang’, and the damage was serious, with an average incidence of 92.87% and a disease index of 81.99. Among them, the occurrence in the Chengdu region was particularly severe, with the incidence and disease index reaching 97.15% and 91.96 respectively. Apart from ‘Hongyang’, the disease also occurred commonly in other red-fleshed varieties, such as ‘Donghong’, with an incidence of 83.63%, but the severity was relatively low, with a disease index of 31.08. The disease occurrences in yellow-fleshed varieties, such as Jinyan and Jinshi, and green-fleshed varieties, such as Hayward, Cuiyu, and *Actinidia arguta* varieties, were relatively low. Pearson correlation analysis showed a significant positive correlation between the incidence and disease index with the cultivar, with the correlation coefficient reaching 0.929 (*p* = 0.002 < 0.01) and 0.795 (*p* = 0.033 < 0.05), respectively (Table 4). The correlation analysis between the occurrence and altitude in the ‘Hongyang’ variety showed that the incidence and disease index were significantly negatively correlated with altitude, with the correlation coefficient reaching −0.780 (*p* = 0.000 < 0.01) and −0.604 (*p* = 0.000 < 0.01), respectively. The incidence and disease index decreased with the increase in altitude.

### 3.2. Screening of Environmental Variable

The initial model was constructed using 67 environmental variables (Table 2), with an AUC value of 0.930. The contribution rate of each variable to the model was calculated, and variables with a contribution rate of less than 1% were excluded through the jackknife test. This process resulted in the selection of 11 variables, including bio3, tmin8, tmin6, prec8, tmax7, prec2, prec4, tavg10, bio2, tmin7, and prec6, with a cumulative contribution rate of 93.2% (Table 5). Pearson correlation analysis among environmental variables revealed high correlations between bio2 and bio3 (correlation coefficient: 0.924) and between tmin7 and tavg10, tmax7, tmin6, and tmin8 (correlation coefficients: 0.924, 0.983, 0.983, and 0.996, respectively), all exceeding 0.90 (Table 6). There will be some problems, such as autocorrelation and multiple linear repetition, and redundant information will be introduced in the process of modeling, which will have an impact on prediction accuracy. To eliminate collinearity between variables and avoid overfitting in the simulation process, bio2 and tmin7 were eliminated. Based on the biological characteristics of kiwifruit brown spot, prec2 was eliminated for low biological significance. In summary, 8 variables, including bio3, prec4, prec6, prec8, tavg10, tmax7, tmin6, and tmin8, were selected to construct the final model.

### 3.3. Suitability Test of MaxEnt Model

**Figure 2:**
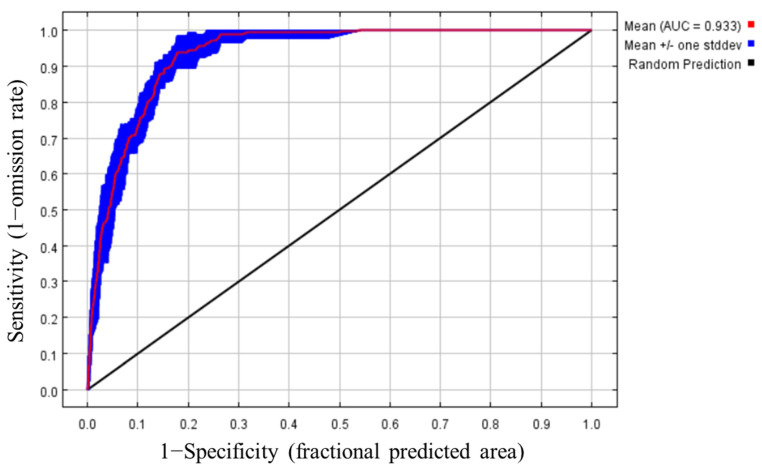
ROC curve analysis and AUC values for the MaxEnt model.

ROC curve analysis of the geographical distribution of kiwifruit brown spot using the MaxEnt model showed that the average AUC value of 10 replicates was 0.933 ± 0.012 (Figure 2), which was significantly higher than the random predicted value of 0.5. According to the evaluation criteria, the accuracy of the model was ‘excellent’. The above results demonstrated that the model had high reliability and could be used for subsequent analysis.

### 3.4. Selection of the Key Environmental Factors

**Figure 3:**
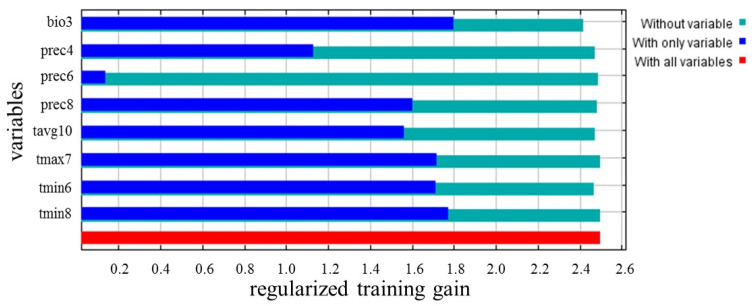
Jackknife test for the importance of environmental variables in the suitability distribution.

The importance of each variable in the final model was determined by examining the regularized training gains when ‘with only variable’, ‘without variable’, and ‘with all variables’ were used for the simulation. As shown in Figure 3, bio3 was identified as the most important factor affecting the distribution of kiwifruit brown spot, and its training gain reached 1.8. tmin8, tmax7, and tmin6 were also important factors, and their individual training gains exceeded 1.7. prec8 and tavg10 were also important for the disease distribution, with training gains of 1.60 and 1.56, respectively. The contribution of prec6 to the model was the lowest. In conclusion, bio3, tmin8, tmax7, tmin6, prec8, and tavg10 were the key environmental factors affecting the distribution of kiwifruit brown spot.

### 3.5. Analysis of Response Curve

**Figure 4:**
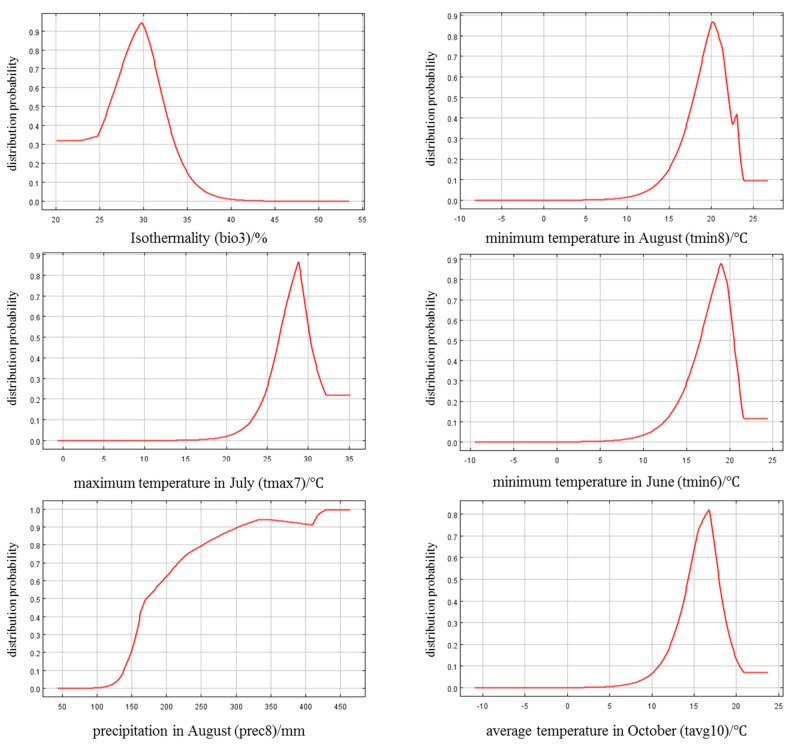
Response curve between distribution probability and environmental variables.

Figure 4 was displays the response curves between the distribution probability and environmental variables, with a probability of 0.33 as the threshold for dividing the suitability of each variable. The results showed that when the suitable range of bio3 was 24.3–33.7%, the distribution probability exceeded 0.33 and reached its highest value at 29.8%, indicating that it was most conducive to the occurrence of kiwifruit brown spot. Low temperatures in August were not conducive to disease occurrence. When tmin8 was below 16.3 °C or above 23.6 °C, the distribution probability was lower than 0.33 and reached its highest value at 20.2 °C. At 22.7–23.1 °C, the probability fluctuated and reached a small peak at 23.0 °C. High temperatures in July were also unfavorable for disease occurrence. When the range of tmax7 was 25.5–29.1 °C, the distribution probability increased with the temperature increasing, and decreased with the temperature increasing at 29.1–31.2 °C. When the range of tmin6 was 15.6–18.8 °C, the distribution probability increased with the temperature increasing, and decreased rapidly at 18.8–20.9 °C. prec8 exceeding 158 mm indicated a rapid occurrence and epidemic of kiwifruit brown spot. The distribution probability reached the highest at 331 and 430 mm, with no further changes when the precipitation exceeded 430 mm. The suitable range of tavg10 was 15.6–18.8 °C, with the peak value at 16.2 °C.

### 3.6. Prediction of Potential Distribution

**Figure 5:**
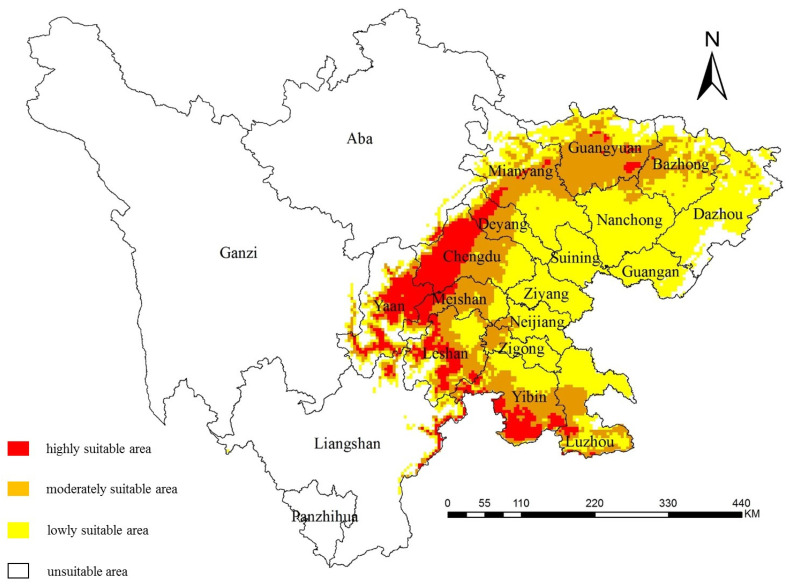
Prediction of the potential distribution of kiwifruit brown spot in Sichuan (city boundary).

According to Figure 5 and Table 7, the highly suitable areas of kiwifruit brown spot in Sichuan were mainly located in the eastern part of the Chengdu region, the central part of the Ya’an region, the southern part of the Yibin region, the central part of Leshan region, and the eastern part of Meishan region, with a total area of 21,849.83 km^2^, accounting for 4.49% of the area of the provincial territory. Among them, the Chengdu and Ya’an regions had the highest proportion of highly suitable areas, with 28.12% and 20.94%, respectively. There were no highly suitable areas in the Dazhou, Ganzi, Guang’an, Nanchong, Neijiang, Panzhihua, Suining, Ziyang, and Zigong regions and no moderately suitable areas in the Guang’an, Panzhihua, Suining, and Ziyang regions. Lowly suitable areas were widely distributed in the whole province, except for the Panzhihua region. The entire Ziyang region was classified as lowly suitable areas. The largest area was occupied by unsuitable areas, reaching 318,185.83 km^2^, mainly distributed in the Ganzi, Aba, and Liangshan regions, with the Ganzi region accounting for the largest proportion, nearly 50%. The entire Panzhihua region was classified as unsuitable areas.

**Figure 6:**
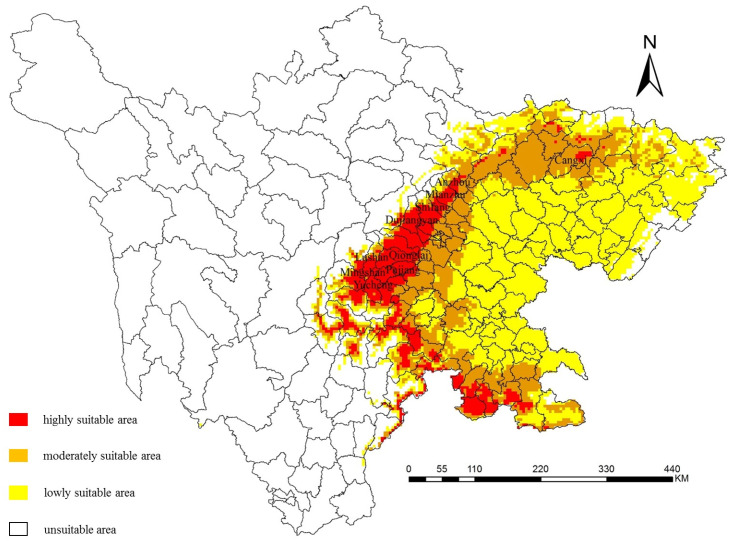
Prediction of the potential distribution of kiwifruit brown spot in Sichuan (country boundary).

According to Figure 6 and Table 8, highly suitable areas were distributed in all eleven central planting areas of kiwifruit. Except for the Anzhou District and Cangxi County, other regions had highly suitable areas accounting for the largest proportion of their total areas. Moreover, the highly suitable area of Qionglai City was the largest, reaching 1348.75 km^2^. More than 90% of the total area in Pujiang County, Qionglai City, Mingshan District, and Yucheng District were classified as highly suitable areas. Among them, Pujiang County and Mingshan District were entirely classified as highly suitable areas. The whole area of the Anzhou District and Cangxi County were mainly moderately suitable areas, accounting for 65.15% and 82.31%, respectively. Mianzhu City had the largest lowly and unsuitable areas in among the eleven regions, covering 197.82 and 261.45 km^2^, respectively.

### 3.7. Field Test of the Model

Out of the 45 test points, 38 of them were accurately simulated (Table 9). The model accuracy calculated was 84.44%, demonstrating high reliability. Among the 7 inaccurate simulation points, all were overfitted, indicating that the level of the predicted suitability was higher than the actual occurrence. 

## 4. Discussion

In this study, continuous monitoring of the epidemic of kiwifruit brown spot in Sichuan revealed that the occurrence of the disease was more severe in the red-fleshed varieties and less severe in the yellow-fleshed and green-fleshed varieties. These findings were consistent with the resistance evaluation of Huang et al. [28] in the kiwifruit germplasm materials to kiwifruit brown spot. The study also highlighted the close relationship between variety layout and the occurrence and epidemic of kiwifruit brown spot. With the continuous expansion of the highly susceptible variety cultivation, kiwifruit brown spot has become the most significant fungal disease in the Sichuan-producing region. However, the impact of variety simplification becomes increasingly evident, and has led to an increased risk of disaster caused by the disease. At present, the prevention of the disease in production is still mainly dependent on chemical control, which is not conducive to the healthy and sustainable development of the kiwifruit industry. The breeding and utilization of resistant varieties are recommended as the most economical and effective measures to control the disease. Therefore, in highly suitable areas, the introduction of resistant varieties, such as Ruiyu and Jinyan, should be recommended. Additionally, the “technical regulations for comprehensive control of kiwifruit brown spot” [31] formulated by Sichuan Agricultural University should be adopted for the scientific and efficient control of the disease. Consistent with the research conclusion of Cui [32], the incidence and disease index were significantly negatively correlated with altitude; as the altitude increased, they decreased gradually. However, further research is needed to determine the altitude boundary for disease occurrence. Since WorldClim datasets are generated by integrating and interpolating the basic data of meteorological stations at different altitudes around the world, altitude information has been implied [18]. Therefore, altitude was not selected as an environmental variable for the MaxEnt prediction model in this study.

Currently, there is limited information on the occurrence and epidemic regularity of kiwifruit brown spot in large-scale areas. Our group has conducted some work on the epidemic dynamics in the early stage, mainly focusing on field disease monitoring and data collection, providing a certain foundation for the construction of prediction models. In this study, the MaxEnt model was employed to simulate and predict the potential distribution of kiwifruit brown spot in Sichuan, and suitability regionalization was conducted using ArcGIS. The suitable areas of each region were calculated, the distribution of central planting areas was analyzed, and the accuracy of the model was verified through field tests. The results indicated that the highly suitable areas of kiwifruit brown spot were mainly located in the Chengdu and Ya’an regions, while the unsuitable areas were mainly distributed in the Ganzi, Aba, and Liangshan regions. The Panzhihua region was entirely classified as an unsuitable area. According to the central planting areas of red-fleshed kiwifruit, Pujiang County and Mingshan District were entirely classified as highly suitable distribution areas. Both kiwifruit brown spot and bacterial canker disease had an adverse impact on the healthy development of the industry. Therefore, in the layout of varieties, both diseases should be comprehensively considered. Ma. [33] demonstrated that the potential severe and suitable areas for kiwifruit bacterial canker disease were mainly distributed along the Longmen Mountains from south to north, connected with the Qinba Mountains, and concentrated in the Ya’an, Chengdu, and Guangyuan regions, which had a high contact ratio with the highly suitable areas for kiwifruit brown spot in this study. Wang et al. [18] indicated that the unsuitable areas of kiwifruit bacterial canker disease in Sichuan were mainly located in the Ganzi, Liangshan, and Panzhihua regions, consistent with the findings of kiwifruit brown spot in this study. The occurrence regionalization of kiwifruit brown spot in Sichuan has been clarified, which not only provided a scientific and effective theoretical basis for the formulation of a prevention and control strategy, but also played an important role in the layout and development of the kiwifruit industry in combination with previous studies on kiwifruit bacterial canker disease.

A disease epidemic is a result of the interaction between host plants and pathogens under the influence of environmental conditions. Environmental conditions mainly include meteorological factors, soil conditions, tillage system, and cultivation measures, with meteorological factors playing an extremely important role. Many researchers have utilized modeling to select key meteorological factors for disease prediction. For instance, Chen et al. [34] found that the amount of rainfall in spring significantly influenced the date of the grapevine downy mildew symptom onset. Chaulagain et al. [35] used correlation analysis and stepwise logistic regression to identify afternoon humid thermal ratio (AHTR), temperature-based duration variables, and their interaction terms as the most significant variables associated with brown rust epidemics of sugarcane in Florida. Kiwifruit brown spot initially occurred in late June, rapidly spread in mid-July, and gradually slowed down until the end of October. In this study, the key environmental variables affecting the potential distribution were identified using the jackknife method, including bio3, tmin8, tmax7, tmin6, prec8, and tavg10, which completely coincide with the actual phenology period of kiwifruit brown spot. Additionally, kiwifruit brown spot develops rapidly under high temperature and humidity conditions, with excessive temperatures inhibiting its development [3,7], which is consistent with the response curve results that the temperature in June and August should not be too low, the temperature in July should not be too high, and the precipitation in August reaches a certain amount. Although our study did not include all factors contributing to the distribution, the selected environmental variables can provide a basis for the future refinement and assessment of a prediction model of kiwifruit brown spot.

An ecological niche model is an emerging technology based on ecological principles, according to the known geographical distribution information of species and corresponding environmental variables, using specific algorithms to calculate the niche demand of target species in the designated area and combining GIS technology to project its distribution probability onto the map [36,37]. However, species distribution models usually lead to overestimating or underestimating the species distribution, namely, false positive and false negative. In this study, the suitability levels predicted by the MaxEnt model were often higher than the actual disease occurrence level, resulting in many false positives. We supposed that the reason for this phenomenon might be attributed to the species distribution model only distinguishing “existence” or “nonexistence” when extracting distribution information, without considering the disease occurrence level, resulting in overfitting. Additionally, the distribution information in this study was mainly obtained through field investigations and local reports inquiry, with a total of 225 distribution points. The data were true, reliable, and relatively systematic, but there might be some omissions in their completeness, which might also cause some errors. Furthermore, previous studies have indicated that the occurrence and epidemic of leaf spot disease were not only affected by meteorological factors, altitude, and cultivars, but also closely related to abiotic factors, such as planting density, site conditions, and canopy density [14,38,39]. Gonzalez-Dominguez et al. [40] developed a model using weather and host phenology to predict the infection period and disease progression of *Phomopsis* cane and leaf spot throughout the season, and validated its performance using ROC analysis (AUROC > 0.7). Ortega-Acosta et al. [41] established a Weibull model using multiple abiotic factors to describe the epidemic dynamics of Roselle leaf and calyx spot induced by *C. cassiicola*. Therefore, various factors should be considered comprehensively in future research to further improve the prediction model of kiwifruit brown spot.

## 5. Conclusions

In this study, the correlation between the occurrence of kiwifruit brown spot and variety and altitude was revealed, its potential distribution was predicted, and the suitable areas were regionalized, which could provide scientific suggestions for the variety layout of kiwifruit in Sichuan. At the same time, six key environmental variables were identified, which could lay the foundation for the subsequent disease prediction and forecast, and provide a theoretical basis for the precise prevention and control of kiwifruit brown spot.

## Figures and Tables

**Table 1:** The standard of classification for kiwifruit brown spot.

Severity	Classification
0	No visible symptoms
1	The spots account for 1–5% of the whole leaf
3	The spots account for 6–25% of the whole leaf
5	The spots account for 26–50% of the whole leaf
7	The spots account for 51–75% of the whole leaf
9	The spots account for above 75% of the whole leaf

**Table 2:** Bioclimatic factors used in the initial model.

Variables	Descriptions	Units
bio1	annual mean temperature	°C
bio2	monthly mean diurnal temperature	°C
bio3	isothermality	%
bio4	standard deviation of seasonal temperature	°C
bio5	max temperature of warmest month	°C
bio6	min temperature of coldest month	°C
bio7	mean temperature annual range	°C
bio8	mean temperature of wettest quarter	°C
bio9	mean temperature of driest quarter	°C
bio10	mean temperature of warmest quarter	°C
bio11	mean temperature of coldest quarter	°C
bio12	annual precipitation	mm
bio13	precipitation of wettest month	mm
bio14	precipitation of driest month	mm
bio15	precipitation variation coefficient	%
bio16	precipitation of wettest quarter	mm
bio17	precipitation of driest quarter	mm
bio18	precipitation of warmest quarter	mm
bio19	precipitation of coldest quarter	mm
prec	precipitation of each month	mm
tavg	average temperature of each month	°C
tmin	min temperature of each month	°C
tmax	max temperature of each month	°C

**Table 3:** Survey information of kiwifruit brown spot.

Region	County	Town	Longitude (E)	Latitude (N)	Altitude (m)	Varieties	Incidence Rate (%)	Disease Index	Occurrence Level
Chengdu	Dujiangyan	Juyuan	103.718199	30.882004	648	Hongyang	100.00	99.45	High
			103.690923	30.923192	624	Hongyang	100.00	99.37	High
			103.685734	30.957274	640	Hongyang	100.00	99.36	High
		Tianma	103.724137	31.019014	622	Hongyang	100.00	99.19	High
			103.737203	31.014943	712	Hongyang	99.47	98.39	High
			103.710977	30.997566	628	Hongyang	99.73	99.17	High
			103.711038	30.997706	622	Hongyang	100.00	99.75	High
			103.717213	31.025650	646	Hongyang	99.87	99.24	High
			103.735692	31.026519	619	Hongyang	100.00	100.00	High
			103.737667	31.026325	687	Hongyang	100.00	99.13	High
			103.609154	30.988223	636	Hongyang	100.00	99.01	High
			103.767738	30.977838	618	Hongyang	100.00	100.00	High
		Puyang	103.676029	31.062330	710	Hongyang	99.07	97.68	High
			103.714189	31.144967	1059	Hongyang	96.80	79.81	High
			103.679077	31.031806	608	Hongyang	100.00	96.99	High
		Shiyang	103.639254	30.888781	618	Hongyang	100.00	96.84	High
			103.649262	30.870515	604	Hongyang	100.00	98.36	High
		Yutang	103.619040	30.970404	733	Hongyang	99.33	96.04	High
		Qingchengshan	103.601324	30.905453	672	Hayward	21.73	2.41	Low
			103.595660	30.859226	601	Hongyang	98.40	94.13	High
		Xingfu	103.678045	30.970588	644	Hongyang	93.47	78.92	High
		Longchi	103.552792	31.057788	912	Hayward	10.40	1.16	Low
			103.664117	31.119242	1234	Hayward	8.40	0.93	Low
	Pujiang	Daxing	103.425850	30.247561	602	Hongyang	100.00	100.00	High
		Ganxi	103.361664	30.263327	597	Hongyang	100.00	100.00	High
		Chengjia	103.402646	30.192967	500	Hongyang/Jinyan	97.73/17.60	96.76/1.96	High
		Dating	103.396489	30.289953	593	Hongyang	100.00	100.00	High
		Xilai	103.510500	30.304396	530	Hongyang	100.00	100.00	High
	Qionglai	Guyi	103.588876	30.390417	479	Hongyang	100.00	96.69	High
		Huojing	103.230814	30.356788	1400	Hongyang	77.20	68.73	High
		Pingle	103.358367	30.383795	547	Hongyang	97.87	95.88	High
		Sangyuan	103.400848	30.546483	622	Hongyang	98.80	94.06	High
		Yangan	103.685419	30.401639	470	Hongyang	100.00	97.78	High
		Datong	103.221808	30.478682	1200	Hongyang	84.27	73.88	High
		Wenjun	103.476656	30.374584	524	Donghong	92.40	47.69	Moderate
	Pengzhou	Lichun	103.897253	31.006937	653	Hongyang	94.53	90.21	High
		Guihua	103.782504	31.102041	830	Hongyang	91.07	83.45	High
		Longmenshan	103.814553	31.260737	1414	Hongyang	84.13	47.34	Moderate
		Bailu	103.919083	31.195717	1128	Hongyang	93.87	86.62	High
		Tongji	103.837542	31.158838	886	Hongyang	96.93	89.17	High
		Gexianshan	103.966427	31.113843	611	Hongyang	93.73	92.13	High
	Xinjin	Xingyi	103.825713	30.456078	200	Hongyang	96.53	62.68	Moderate
		Huaqiao	103.869465	30.431029	462	Hongyang	87.60	60.55	Moderate
	Dayi	Xinchang	103.451827	30.525427	561	Hongyang	98.93	96.90	High
		Wangsi	103.521293	30.527021	521	Hongyang	100.00	97.34	High
		Yuelai	103.442643	30.631438	640	Hongyang	98.40	94.13	High
		Anren	103.590849	30.467129	494	Hongyang	99.73	99.01	High
Ya’an	Yucheng	Shangli	103.039111	30.158470	889	Hongyang	91.47	87.05	High
			103.027521	30.163007	906	Yidun	19.47	2.65	Low
		Bifengxia	103.018498	30.109764	984	Hongyang	91.60	72.16	High
		Caoba	103.140857	29.996113	600	*A. arguta*	12.93	1.44	Low
		Duoying	102.916580	30.020083	754		3.73	0.41	Low
		Daxing	102.986775	29.954752	688	Hongyang	93.87	87.01	High
		Babu	102.909216	29.883380	600	*A. arguta*	9.20	1.02	Low
	Mingshan	Yongxing	103.158857	30.042103	596	Hongyang	100.00	96.99	High
			103.143947	30.049887	549	Hongshi	52.80	7.01	Low
		Jianshan	103.117475	30.157528	766	Hongyang	99.20	96.93	High
		Mengdingshan	103.072239	30.108014	774	Donghong	94.13	27.12	Low
		Maling	103.324317	30.129206	684	Hongyang	100.00	94.62	High
		Maohe	103.369168	30.219322	600	Hongyang	97.73	90.33	High
		Heizhu	103.245720	30.247830	670	Gold 3	6.80	0.76	Low
		Zhongfeng	103.181129	30.188253	756	Hongyang	95.20	90.92	High
		Mengyang	103.116365	30.114172	701	Hongyang	93.07	91.57	High
		Wangu	103.128377	30.170035	674	Hongyang	93.47	88.38	High
			103.137349	30.185314	679	Hongyang	91.73	93.66	High
			103.130274	30.141192	890	Hongyang	91.07	88.67	High
		Baizhan	103.311937	30.188603	624	Hongyang	85.73	87.30	High
			103.261842	30.191358	681	Hongyang	70.53	64.56	High
	Yingjing	Wuxianxiang	102.845183	29.780242	936	Hongyang	89.20	67.01	High
		Huatan	102.786623	29.778432	935	Hongyang	84.67	66.83	High
			102.780811	29.783500	875	Hongyang	91.73	74.12	High
		Yandao	102.850645	29.820925	894	Hongyang	90.27	73.93	High
		Qinglong	102.868851	29.769872	722	Hongyang	94.27	75.60	High
			102.881259	29.773278	1529	Hongyang	70.13	67.24	High
		Anjing	102.761545	29.737654	868	Hongyang	89.73	51.91	Moderate
		Longcanggou	102.846983	29.707985	909	Hongyang	89.20	48.90	Moderate
		Siping	102.669532	29.780159	1066	Cuiyu	14.40	1.60	Low
		Baofeng	102.826392	29.863237	949	Hongyang	85.47	71.54	High
		Xintian	102.856832	29.828373	894		7.07	0.79	Low
	Lushan	Feixianguan	102.915150	30.093092	666	Hongyang	100.00	97.02	High
		Luyang	102.960005	30.157746	965	Hongyang	85.07	71.39	High
		Siyan	102.910364	30.142439	642	Hongyang	99.20	95.27	High
		Longmen	103.025524	30.260615	630	Hongyang	97.20	89.32	High
	Shimian	Meiluo	102.438117	29.315473	1335	Jinyan	13.73	1.53	Low
		Yingzheng	102.415574	29.274978	1388	Hongyang	87.07	69.59	High
			102.411492	29.277706	1420	Hongyang	80.67	67.90	High
			102.410712	29.271969	1369	Hongyang	78.27	68.19	High
		Xinmin	102.195943	29.412436	1519	Hongyang	86.67	65.34	Moderate
		Xieluo	102.180677	29.219755	1243	Hongyang	84.40	69.13	high
			102.182687	29.211921	1294	Hongyang	78.13	44.44	Moderate
			102.255037	29.213112	1585	Hongyang	76.80	39.81	Moderate
		Caoke	102.122809	29.404204	1357	Hongyang	72.93	38.93	Moderate
			102.086688	29.390256	1538	Hongshi 2	57.47	10.83	Low
		Anshunchang	102.284151	29.259668	1240	Hongyang	82.93	69.99	High
	Baoxing	Daxi	102.943342	30.553705	741	Hongyang	93.86	63.88	Moderate
		Muping	102.803471	30.347224	869	Hongyang	86.53	59.54	Moderate
		Longdong	102.679432	30.464322	1258	Hongyang	78.40	45.44	Moderate
			102.720493	30.494849	1758	Hongyang	66.26	41.50	Moderate
Guangyuan	Cangxi	Dongxi	106.282834	32.044742	749	Hongyang	97.07	94.15	High
		Longshan	106.332543	31.885837	630	Hongyang	94.80	80.47	High
		Yuedong	106.239293	31.959870	700	Hongyang	87.47	78.68	High
		Yuanba	106.090342	31.860973	400	Hongyang	96.27	62.83	Moderate
		Qiping	106.132578	31.898718	430	Hongyang	92.80	59.75	Moderate
		Wenchang	106.363683	31.987743	655	Hongyang	94.67	77.50	High
		Lingjiang	105.960366	31.750199	400	Hongyang	93.73	66.12	Moderate
		Longwang	105.982809	31.984019	650	Hongyang	91.60	79.81	High
	Zhaohua	Weizi	105.895860	32.174634	709	Hongyang	93.07	92.09	High
		Yuanba	105.973514	32.329941	597	Hongyang	97.20	94.62	High
		Zhaohua	105.723692	32.337770	626	Hongyang	90.53	66.34	Moderate
Deyang	Shifang	Jiandi	104.039288	31.224755	717	Hongshi/Jinshi	39.47/5.73	6.76/0.64	Low
		Yinghua	104.027888	31.297691	916	Hongyang	85.87	69.08	High
	Mianzhu	Jiulong	104.139889	31.411938	969	Hongshi/Jinshi	44.00/3.87	8.69/0.43	Low
		Guangji	104.115350	31.257515	617	Hongshi/Jinshi	38.13/2.27	8.19/0.25	Low
		Yuquan	104.132120	31.257500	582	Donghong/Huapu	70.80/13.73	14.61/1.53	Low
		Xiaode	104.239352	31.241509	530	Donghong	77.20	34.89	Moderate
Mianyang	Anzhou	Huangtu	104.435962	31.545840	585	Hongyang	100.00	93.76	High
		Sangzao	104.333649	31.593687	661	Hongyang	100.00	97.11	High
	Beichuan	Yongchang	104.440374	31.582567	600	Hongyang	100.00	87.05	High
		Guixi	104.647893	31.987493	893	Hongyang	79.07	49.20	Moderate
		Tongquan	104.608754	31.770263	722	Hongyang	93.73	83.45	High
Meishan	Pengshan	Xiejia	103.706150	30.262840	550	Hongyang	99.20	94.06	High
	Dongpo	Funiu	103.942803	30.089608	471	Hongyang	94.67	59.27	Moderate

**Table 4:** Pearson correlation analysis between the occurrence of kiwifruit brown spot and varieties and altitude.

	Incidence Rate		Disease Index	
	Correlation Coefficient	Significance	Correlation Coefficient	Significance
varieties	0.929 **	0.002	0.795 *	0.033
altitude	−0.780 **	0.000	−0.604 **	0.000

Note: * and ** indicate *p* < 0.05 and 0.01, respectively.

**Table 5:** The accumulated contribution of each environmental variable to the potential distribution of kiwifruit brown spot.

Variables	Contribution (%)	Cumulative (%)
bio3	25.8	25.8
tmin8	20.9	46.7
tmin6	17.0	63.7
prec8	16.7	80.4
tmax7	3.1	83.5
prec2	2.2	85.7
prec4	2.1	87.8
tavg10	1.5	89.3
bio2	1.4	90.7
tmin7	1.4	92.1
prec6	1.1	93.2

**Table 6:** Pearson correlation analysis of environmental variables affecting the distribution of kiwifruit brown spot.

	bio2	bio3	prec2	prec4	prec6	prec8	tavg10	tmax7	tmin6	tmin7
bio3	0.924									
prec2	−0.370	−0.154								
prec4	−0.475	−0.524	0.501							
prec6	−0.321	−0.031	0.705	0.427						
prec8	0.080	0.257	0.664	0.100	0.327					
tavg10	−0.613	−0.555	0.415	0.216	0.101	0.288				
tmax7	−0.705	−0.767	0.249	0.427	0.011	0.008	0.897			
tmin6	−0.688	−0.730	0.244	0.301	−0.086	0.136	0.942	0.958		
tmin7	−0.766	−0.797	0.290	0.398	0.025	0.074	0.924	0.983	0.983	
tmin8	−0.781	−0.806	0.296	0.398	0.041	0.077	0.918	0.977	0.980	0.996

**Table 7:** Prediction of the potential distribution area of kiwifruit brown spot (regions).

Region	Unsuitable Area		Lowly Suitable Area		Moderately Suitable Area		Highly Suitable Area	
	Area (km^2^)	Proportion (%)	Area (km^2^)	Proportion (%)	Area (km^2^)	Proportion (%)	Area (km^2^)	Proportion (%)
Aba	84,075.16	26.42	672.30	0.70	205.43	0.41	168.08	0.77
Bazhong	1550.03	0.49	7152.55	7.47	3884.41	7.73	18.68	0.09
Chengdu	747.00	0.23	2931.99	3.06	4594.07	9.14	6144.10	28.12
Dazhou	3828.39	1.20	12,381.57	12.94	448.20	0.89	0.00	0.00
Deyang	336.15	0.11	2670.53	2.79	2241.01	4.46	877.73	4.02
Ganzi	149,157.78	46.88	373.50	0.39	280.13	0.56	0.00	0.00
Guang’an	653.63	0.21	5509.15	5.76	0.00	0.00	0.00	0.00
Guangyuan	1848.83	0.58	4519.37	4.72	9412.23	18.73	597.60	2.74
Leshan	1624.73	0.51	3735.01	3.90	4594.07	9.14	2801.26	12.82
Liangshan	55,203.50	17.35	1998.23	2.09	989.78	1.97	859.05	3.93
Luzhou	298.80	0.09	7376.65	7.71	3716.34	7.40	392.18	1.79
Meishan	448.20	0.14	1755.46	1.83	3678.99	7.32	1344.60	6.15
Mianyang	5303.72	1.67	9057.41	9.46	5714.57	11.37	541.58	2.48
Nanchong	0.00	0.00	11,690.59	12.21	1064.48	2.12	0.00	0.00
Neijiang	0.00	0.00	4911.54	5.13	466.88	0.93	0.00	0.00
Panzhihua	7245.93	2.28	0.00	0.00	0.00	0.00	0.00	0.00
Suining	0.00	0.00	5322.39	5.56	0.00	0.00	0.00	0.00
Ya’an	5863.97	1.84	2334.38	2.44	2297.03	4.57	4575.39	20.94
Yibin	0.00	0.00	3959.11	4.14	5602.52	11.15	3529.59	16.15
Ziyang	0.00	0.00	5733.25	5.99	0.00	0.00	0.00	0.00
Zigong	0.00	0.00	3305.49	3.45	1064.48	2.12	0.00	0.00
Total	318,185.83	100.00	95,709.73	100.00	50,254.61	100.00	21,849.83	100.00

**Table 8:** Prediction of the potential distribution area of kiwifruit brown spot (central planting areas).

Region	Country (District)	Unsuitable Area		Lowly Suitable Area		Moderately Suitable Area		Highly Suitable Area	
		Area (km^2^)	Proportion of Whole Area (%)	Area (km^2^)	Proportion of Whole Area (%)	Area (km^2^)	Proportion of Whole Area (%)	Area (km^2^)	Proportion of Whole Area (%)
Chengdu	Dujiang	186.75	15.87	53.95	4.76	89.92	7.94	1132.95	71.43
	Pujiang	0.00	0.00	0.00	0.00	0.00	0.00	629.42	100.00
	Qionglai	0.00	0.00	0.00	0.00	89.92	6.67	1348.75	93.33
Deyang	Mianzhu	261.45	20.59	197.82	16.18	251.77	20.59	1222.87	42.65
	Shifang	130.73	15.56	53.95	6.67	305.72	37.78	809.25	40.00
Guangyuan	Cangxi	0.00	0.00	107.90	4.62	1924.22	82.31	317.48	13.08
Mianyang	Anzhou	56.03	4.55	89.92	7.58	773.29	65.15	1186.90	22.73
Ya’an	Lushan	205.43	17.19	89.92	7.81	305.72	26.56	1150.94	48.44
	Mingshan	0.00	0.00	0.00	0.00	0.00	0.00	593.45	100.00
	Yucheng	0.00	0.00	0.00	0.00	71.93	6.78	1061.02	93.22

**Table 9:** Detailed information of the actual survey points for the field test.

Region	Country	Town	Longitude	Latitude	Altitude (m)	Varieties	Incidence Rate (%)	Disease Index	Occurrence Level	Suitability Level
Chengdu	Dujiangyan	Tianma	103.729050	31.032388	601	Hongyang	100	97.53	High	High
			103.743516	31.025524	600	Hongyang	100	97.69	High	High
			103.756496	31.020132	621	Hongyang	100	98.76	High	High
		Juyuan	103.657528	30.949170	653	Hongyang	100	93.82	High	High
	Pujiang	Fuxing	103.443221	30.317316	538	Hongyang	98.13	90.77	High	High
			103.438191	30.321275	546	Hongyang	98.8	90.18	High	High
			103.436934	30.325817	528	Hongyang	97.87	86.86	High	High
		Datang	103.418606	30.299636	587	Hongyang	94.93	88.53	High	High
		Daxing	103.397353	30.235533	569	Hongyang	96.13	90.03	High	High
	Qionglai	Sangyuan	103.469178	30.461789	517	Hongyang/Donghong	93.60/73.07	89.77/16.39	High	High
		Kongming	103.466594	30.342379	607	Hongyang	93.07	88.73	High	High
		Guyi	103.516878	30.373997	586	Hongyang	93.87	93.17	High	High
		Baolin	103.517850	30.331534	604	Hongyang	91.87	95.97	High	High
			103.516878	30.373990	589	Hongyang	94.8	89.44	High	High
	Chongzhou	Qiquan	103.641074	30.563695	438	Hongyang/Donghong	100/77.47	98.36/31.73	High	High
			103.660735	30.560634	451	Hongyang/Donghong	100/85.60	97.60/27.76	High	High
Ya’an	Yucheng	Shangli	103.059266	30.152868	963	*A. arguta*	12.13	1.26	Low	High
			103.070186	30.159260	968	Gold 3	5.73	0.64	Low	High
		Duoying	102.914164	30.015172	587	Hongyang	89.87	93.64	High	High
	Baoxing	Muping	102.832564	30.408815	1104	Hongyang	82.8	70.53	High	High
	Yingjing	Qinglong	102.877242	29.767267	1091	Hongyang/Chuanmi	84.87/37.86	74.49/25.21	High	High
	Shimian	Anshunchang	102.258098	29.310755	1399	Hongyang	82.93	72.55	High	High
Guangyuan	Cangxi	Tingzi	105.859244	31.819290	449	Hongyang	93.6	74.4	High	High
		Yunfeng	106.004130	31.715040	490	Hongyang	90.4	72.09	High	High
			106.002725	31.724609	580	Hongyang	87.87	68.98	High	High
			106.018840	31.879743	519	Hongyang/Jinhong	88.80/32.40	66.85/15.45	High	High
		Lingjiang	105.977369	31.799844	696	Hongyang	84.13	61.05	Moderate	Moderate
		Yongning	106.002692	31.724439	588	Hongyang	81.07	64.03	Moderate	Moderate
			105.916557	32.002183	699	Hongyang	78.53	59.82	Moderate	Moderate
		Longwang	105.959645	31.981199	681	Hongyang	79.87	62.39	Moderate	Moderate
	Jiange	Puan	105.449204	32.127941	704	Hongyang	74.13	54.56	Moderate	Moderate
			105.422221	32.008581	686	Hongyang	77.47	56.12	Moderate	Moderate
		Longyuan	105.450482	31.986242	671	Hongyang	72.8	52.83	Moderate	Moderate
	Qingchuan	Zhuyuan	105.355354	32.249336	567	Hongyang	76.13	45.14	Moderate	Moderate
Deyang	Mianzhu	Guangji	104.069418	31.260198	661	Hongyang	94.4	81.97	High	High
		Jiulong	104.126292	31.380313	701	Hongyang	91.73	76.93	High	High
			104.124498	31.390861	716	Gold 3	2.93	0.33	Low	High
Mianyang	Anzhou	Huangtu	104.435304	31.545761	578	Hongyang	100	97.09	High	High
			104.425117	31.569665	627	Hongyang	100	98.27	High	High
			104.440072	31.551297	635	Hongyang	100	93.66	High	High
Leshan	Mabian	Laodong	103.562148	28.946852	1023	Jinhong	16.4	1.82	Low	High
			103.575287	28.938294	1022	Gold 3	7.87	0.87	Low	High
Luzhou	Xuyong	Huangni	105.348891	28.004857	1006	Gold 3	11.6	1.29	Low	High
	Gulin	Dongxing	106.090053	27.984000	1180	Guichang	13.73	1.52	Low	Moderate
		Yonghe	105.915008	28.067051	463		16.53	1.84	Low	Low

## Data Availability

The data presented in this study are available on request from the corresponding author.
